# Association of red cell distribution width with the risk of 3-month readmission in patients with heart failure: A retrospective cohort study

**DOI:** 10.3389/fcvm.2023.1123905

**Published:** 2023-03-07

**Authors:** Fang Gu, Han Wu, Xiaoli Jin, Cheng Kong, Wenyan Zhao

**Affiliations:** ^1^ Center for Reproductive Medicine, Department of Pediatrics, Zhejiang Provincial People’s Hospital, Affiliated People’s Hospital, Hangzhou Medical College, Hangzhou, China; ^2^ Department of Clinical Laboratory Medicine, Sir Run Run Shaw Hospital Xiasha Campus, Zhejiang University School of Medicine, Hangzhou, China; ^3^ Department of Neurosurgery, People’s Hospital of Pan’an County, Jinhua, China; ^4^ Center for General Practice Medicine, Department of General Practice Medicine, Zhejiang Provincial People’s Hospital, Affiliated People’s Hospital, Hangzhou Medical College, Hangzhou, China

**Keywords:** red cell distribution width (RDW), prognosis, readmission, heart failure, China

## Abstract

**Background:**

In recent years, red cell distribution width (RDW) has been found to be associated with the prognosis of patients with heart failure (HF) in Western countries. However, evidence from Asia is limited. We aimed to investigate the relationship between RDW and the risk of 3-month readmission in hospitalized Chinese HF patients.

**Methods:**

We retrospectively analyzed HF data from the Fourth Hospital of Zigong, Sichuan, China, involving 1,978 patients admitted for HF between December 2016 and June 2019. The independent variable in our study was RDW, and the endpoint was the risk of readmission within 3 months. This study mainly used a multivariable Cox proportional hazards regression analysis. Smoothed curve ﬁtting was then used to assess the dose-response relationship between RDW and the risk of 3-month readmission.

**Results:**

In the original cohort of 1,978 patients with HF (42% male and 73.1% aged ≥70 years), 495 patients (25.0%) were readmitted within 3 months after discharge. Smoothed curve ﬁtting showed a linear correlation between RDW and the risk of readmission within 3 months. In the multivariable-adjusted model, every 1% increase in RDW was associated with a 9% increased risk of readmission within 3 months (hazard ratio = 1.09, 95% confidence interval: 1.00–1.15; *P* < 0.005).

**Conclusions:**

A higher RDW value was significantly associated with a greater risk of 3-months readmission in hospitalized patients with HF.

## 1. Introduction

Heart failure (HF) is one of the major global public health issues and a common cause of hospitalization in middle-aged and older adults (1). Traditional and innovative therapies have been used to treat and manage HF, leading to a significant reduction in HF mortality (2, 3). However, the high rate of readmission of HF patients within 3 months of discharge is an emerging problem that can cause a clinical and economic burden. Readmission of HF patients within 3 months after discharge may be seen as a warning sign of a worse subsequent prognosis for the patient. A study from Japan concluded that patients with HF who were readmitted within 3 months had a higher risk of death at long-term follow-up (4); therefore, early identification of this group of patients with HF could optimize treatment more quickly, improve symptoms, increase patient survival, reduce readmission rates, and reduce the economic burden on families and society.

Several variables have been associated with readmission in patients with HF. Apostolos et al. conducted a propensity score-matched observational study that included HF patients with ejection fraction use from the U.S. Medicare-related OPTIMIZE-HF registry from March 2003 to December 2004 and found that systolic blood pressure (SBP) <120 mmHg at discharge was associated with an increased risk of readmission in these patients (5). However, this study included only patients with HF with an ejection fraction of ≥50%; therefore, it is not possible to infer whether an SBP of <120 mmHg at discharge is associated with an increased risk of readmission in all HF patients. Ioanna et al. also studied 671 HF patients from the OPTIMIZE-HF registry (6). They found that psychosocial factors were associated with 1-year readmission risk in HF patients. Still, their study sample size was small, and the questionnaire for assessing psychosocial factors was too cumbersome and complex for clinical replication. Therefore, we need to find an earlier and easier indicator to detect the risk of readmission and better manage this group of inpatients with HF.

Red cell distribution width (RDW), an indicator of changes in red blood cell (RBC) size and shape, is easily obtained from a complete blood count and is a simple, inexpensive measure (7). The RDW typically ranges from 11% to 15%, and a higher RDW value indicates greater variability in size (8). Traditionally, RDW has been used to differentiate the cause of anemia (9). Recent clinical evidence suggests that changes in RBC size are associated with the development and adverse outcomes of non-hematologic diseases, such as stroke, osteoporosis, dementia, and cardiovascular disease (10–13). Especially in patients with HF, RDW has been shown to be associated with first hospitalization and poor prognosis in HF (14, 15). However, most of the available evidence is focused on the United States and Europe, and there are few studies related to Asia, especially China.

Thus, this study aimed to investigate the relationship between RDW and the risk of 3-month readmission in hospitalized Chinese HF patients.

## 2. Materials and methods

### 2.1. Study population

This study used a single-center database included in PhysioNet (16). This dataset collected information on a total of 2,008 adult HF patients from December 2016 to June 2019 at the Fourth People's Hospital in Zigong, Sichuan, China, to understand the characteristics of the Chinese HF population (17). The data collected in this dataset included demographic data, baseline clinical characteristics, co-morbidities, laboratory test results, medications, and outcomes. This study followed the STROBE (Strengthening the Reporting of Observational Studies in Epidemiology) statement and was designed to investigate whether elevated RDW is correlated with an increased risk of rehospitalization. The Ethics Committee of the Fourth People's Hospital of Zigong approved this study (approval number:2020-010). The requirement for informed consent was waived owing to the retrospective design of this study. This study was conducted in strict accordance with the Declaration of Helsinki. HF was defined according to the criteria of the European Society of Cardiology (18).

### 2.2. Study variables

In this study, the exposure variable was RDW, and the primary outcome variable was the risk of readmission within 3 months. We also collected additional data from the database, including age, sex, Charlson Comorbidity Index (CCI) score, New York Heart Association (NYHA) cardiac function classification, type of HF (right, left, both), systolic blood pressure, diastolic blood pressure, body mass index, brain natriuretic peptide, white blood cell count, RBC count, hematocrit, hemoglobin, mean cell volume, mean cell hemoglbin content, mean cell hemoglobin concentration, platelet, total protein, albumin, alanine aminotransferase, aspartate transaminase, lactate dehydrogenase, alkaline phosphatase, γ-glutamyltranspeptidase, and estimated glomerular filtration rate (eGFR, ml/min per 1.73 m^2^)).

### 2.3. Statistical analysis

Categorical variables were analyzed using percentages, and normally continuous variables were expressed as mean ± standard deviation. Creatinine levels were included in the Chronic Kidney Disease Epidemiology Collaborative Study formula to estimate the glomerular filtration rate. The patients were divided into three groups according to the tertiles of RDW. First, this study used linear regression models and *χ*^2^ tests to compare the baseline characteristics of the patients in different groups. Second, univariate and multivariate Cox proportional hazards regression analyses were applied to estimate the correlation between RDW and the probability of readmission within 3 months. Three models were simultaneously calculated as follows: a non-adjusted model, not adjusted for potential confounders; a minimally adjusted model, adjusted for age and sex; a multivariable-adjusted model, adjusted for confounders in the minimally adjusted model + body mass index, CCI score, NYHA cardiac function classification, type of HF, brain natriuretic peptide, RBC, HB, total protein, albumin, alanine aminotransferase, lactate dehydrogenase, and eGFR. The covariates selected for adjustment were based on the fact that their addition to the model changed the regression coefficient by at least 10%. 95% confidence intervals (CIs) and hazard ratios (HRs) were estimated for all models. Third, sensitivity analyses were conducted in this study to test the robustness of the results. The RDW was converted into a categorical tertile variable. The *P* for the trend was calculated. We examined whether the results were consistent with those of RDW as a continuous variable. Stratiﬁed analyses and interactions were implemented according to age, sex, CCI score, NYHA cardiac function classification, HF type, eGFR, and absolute iron deficiency. Absolute iron deficiency referred to an RDW > 15% with: either a mean cell volume <80 fl or a mean cell hemoglobin content <27 pg or a mean cell hemoglobin concentration <32 g/dl (19).

We used smooth curve ﬁttings (penalized spline method) to evaluate the dose-response relationship between RDW and the risk of 3-month readmission. A cumulative 3-month readmission-free probability analysis was performed using Kaplan–Meier curves with log-rank statistics according to the different groups.

All tests were two-sided, and *P* < 0.05 was considered statistically significant. Data were analyzed using the R statistical package (R Foundation for Statistical Computing, Vienna, Austria; http://www.r-project.org; version 3.6.3) and Free Statistics software version 1.7.

## 3. Results

### 3.1. Patient selection

The database included 2008 patients with HF, 30 of whom were excluded because of missing RDW values on admission. Therefore, 1,978 patients were included in the analysis.

### 3.2. Baseline characteristics

The selected patient characteristics are shown in Table 1. Of these patients, 73.1% were older than 70 years, and 42% were male. Compared with the patients in the lower RDW groups (tertile 1 and tertile 2), patients with a higher RDW (tertile 3) were more likely to be male, have total HF, have higher CCI scores, NYHA class, brain natriuretic peptide, lactate dehydrogenase, alkaline phosphatase, γ-glutamyltranspeptidase, and have lower body mass index, RBC count, mean cell volume, mean cell hemoglobin content, mean cell hemoglobin concentration, hematocrit, HB, total protein, albumin, and eGFR (*P* < 0.01).

**Table 1:** Baseline characteristics of the participants.

Characteristics	Red cell distribution width (%)				*P*-value
	Total	Tertile1 (11.8–13.8)	Tertile 2 (13.9–15.0)	Tertile 3 (15.1–29.9)	
	*n* = 1,978	*n* = 634	*n* = 669	*n* = 675	
Age, year					<0.001
<70	533 (26.9%)	207 (32.6%)	158 (23.6%)	168 (24.9%)	
≥70	1,445 (73.1%)	427 (67.4%)	511 (76.4%)	507 (75.1%)	
Male	830 (42.0%)	267 (42.1%)	276 (41.3%)	287 (42.5%)	
CCI score					0.074
<3	1,503 (76.2%)	491 (77.9%)	519 (77.6%)	493 (73.1%)	
≥3	470 (23.8%)	139 (22.1%)	150 (22.4%)	181 (26.9%)	
NYHA cardiac function classification					<0.001
Class II	341 (17.2%)	124 (19.6%)	113 (16.9%)	104 (15.4%)	
Class III	1,027 (51.9%)	357 (56.3%)	337 (50.4%)	333 (49.3%)	
Class IV	610 (30.8%)	153 (24.1%)	219 (32.7%)	238 (35.3%)	
Type of heart failure					0.004
Right	50 (2.5%)	17 (2.7%)	20 (3.0%)	13 (1.9%)	
Left	464 (23.5%)	169 (26.7%)	168 (25.1%)	127 (18.8%)	
Both	1,464 (74.0%)	448 (70.7%)	481 (71.9%)	535 (79.3%)	
SBP, mmHg	131.2 ± 24.8	132.8 ± 23.9	131.2 ± 24.8	129.6 ± 25.4	0.058
DBP, mmHg	76.6 ± 14.5	76.6 ± 14.1	77.3 ± 14.7	75.8 ± 14.7	0.172
BMI, kg/m2	21.8 ± 13.7	23.2 ± 23.1	21.2 ± 6.0	21.1 ± 3.9	0.006
BNP, pg/ml	1,285.2 ± 1,353.4	953.3 ± 1,201.3	1,321.4 ± 1,308.8	1,562.9 ± 1,463.1	<0.001
WBC, 10^9^/L	7.3 ± 3.5	7.5 ± 3.3	7.4 ± 3.3	7.1 ± 3.8	0.076
RBC, 10^12^/L	3.9 ± 0.8	3.9 ± 0.6	3.9 ± 0.7	3.8 ± 0.9	<0.001
MCH, pg	29.9 ± 3.4	31.5 ± 1.8	30.6 ± 2.5	27.8 ± 4.2	<0.001
MCV, fl	92.0 ± 8.8	94.9 ± 4.9	93.6 ± 7.1	87.8 ± 11.2	<0.001
MCHC, g/L	324.8 ± 14.0	332.2 ± 10.0	326.8 ± 10.2	316.0 ± 15.6	<0.001
HCT, %	0.4 ± 0.1	0.4 ± 0.1	0.4 ± 0.1	0.3 ± 0.1	<0.001
HB, g/L	115.1 ± 24.5	122.9 ± 19.9	119.3 ± 21.6	103.6 ± 26.9	<0.001
PLT, 10^9^/L	145.2 ± 65.0	150.7 ± 57.2	140.9 ± 61.1	144.3 ± 74.6	0.021
Total protein, g/L	65.1 ± 7.4	65.8 ± 6.7	64.9 ± 7.0	64.6 ± 8.2	0.007
ALB, g/L	36.5 ± 5.0	37.7 ± 4.6	36.9 ± 4.7	35.0 ± 5.2	<0.001
ALT, U/L	54.2 ± 205.2	43.8 ± 166.3	56.1 ± 237.8	62.1 ± 202.8	0.278
AST, U/L	63.6 ± 299.2	52.1 ± 284.0	52.6 ± 168.7	85.9 ± 399.3	0.088
LDH, IU/L	274.1 ± 254.6	247.6 ± 175.5	260.8 ± 161.4	313.4 ± 367.5	<0.001
ALP, U/L	89.6 ± 45.2	81.6 ± 33.7	89.3 ± 37.2	97.3 ± 58.6	<0.001
GGT, U/L	61.3 ± 68.8	52.8 ± 53.0	64.5 ± 69.6	65.9 ± 79.6	0.001
eGFR, ml/min/1.73 m^2^	68.6 ± 36.7	73.3 ± 36.8	67.6 ± 34.6	65.2 ± 38.0	<0.001

CCI, charlson comorbidity index; NYHA, new york heart association; SBP, systolic blood pressure; DBP, diastolic blood pressure; BMI, body mass index; BNP, brain natriuretic peptide; WBC, white blood cell; RBC, red blood cell; MCV, mean cell volume; MCHC, mean cell hemoglobin concentration; MCH, mean cell hemoglobin content; HCT, hematocrit; HB, hemoglobin; PLT, platelet; ALB, albumin; ALT, alanine aminotransferase; AST, aspartate transaminase; LDH, lactate dehydrogenase; ALP, alkaline phosphatase; GGT, γ-glutamyltranspeptidase; eGFR, estimated glomerular filtrationrate.

### 3.3. Association between RDW and the risk of readmission within 3 months

Univariate analysis suggested that age, CCI score, cardiac function class, NYHA cardiac function classification, systolic blood pressure, diastolic blood pressure, RBC, hematocrit, HB, eGFR, and RDW were associated with readmission risk within 3 months (*P* < 0.05) (Table 2).

**Table 2:** Results of the univariate analysis.

Covariate	HR (95% CI)	*P*-value
Age, year		
<70	Ref	
≥70	1.299 (1.053, 1.604)	0.015
Sex		
Female	Ref	
Male	1.051 (0.880, 1.256)	0.582
CCI score		
<3	Ref	
≥3	1.495 (1.234, 1.812)	0.000
NYHA cardiac function classification		
Class II	Ref	
Class III	1.496 (1.124, 1.990)	0.006
Class IV	1.947 (1.450, 2.615)	<0.001
Type of heart failure		
Right	Ref	
Left	0.885 (0.459, 1.706)	0.715
Both	1.460 (0.780, 2.735)	0.237
SBP	0.991 (0.987, 0.994)	<0.001
DBP	0.992 (0.985, 0.998)	0.007
BMI	1.001 (0.994, 1.007)	0.831
BNP	1.000 (1.000, 1.000)	0.002
WBC	0.992 (0.966, 1.018)	0.523
RBC	0.837 (0.747, 0.938)	0.002
HCT	0.191 (0.055, 0.664)	0.009
HB	0.996 (0.992, 0.999)	0.023
PLT	0.999 (0.998, 1.001)	0.456
Total protein	0.995 (0.983, 1.008)	0.448
ALB	1.003 (0.985, 1.022)	0.750
ALT	1.000 (0.999, 1.000)	0.420
AST	1.000 (0.999, 1.000)	0.528
LDH	1.000 (1.000, 1.000)	0.690
ALP	1.001 (0.999, 1.003)	0.311
GGT	1.001 (0.999, 1.002)	0.341
eGFR	0.995 (0.992, 0.998)	0.000
RDW	1.065 (1.024, 1.107)	0.001

HR, hazard ratio; CI, confidence interval; Ref., reference; CCI, charlson comorbidity index; NYHA, new york heart association; SBP, systolic blood pressure; DBP, diastole blood pressure; BMI, body mass index; BNP, brain natriuretic peptide; WBC, white blood cell; RBC, red blood cell; HCT, hematocrit; HB, hemoglobin; PLT, platelet; ALB, albumin; ALT, alanine aminotransferase; AST, aspartate transaminase; LDH, lactate dehydrogenase; ALP, alkaline phosphatase; GGT, *γ*-glutamyltranspeptidase; eGFR, estimated glomerular filtrationrate; RDW, red cell distribution width.

Table 3 shows that 495 (25.0%) participants were readmitted within 3 months. In the RDW tertile 1–3 groups, 132 (20.8%), 175 (26.2%), and 188 (27.9%) patients with HF, respectively, were readmitted within 3 months. The results of the multivariable Cox proportional hazards regression analysis suggested that an elevated RDW was associated with an increased risk of readmission within 3 months. In the multivariable-adjusted model, every 1% increase in RDW was associated with a 9% increased risk of readmission within 3 months (HR = 1.09, 95% CI: 1.00–1.15, *P* = 0.003). We also analyzed RDW as a categorical variable one more time. Compared with participants with a RDW lower than 13.8% (tertile 1), the probability of readmission within 3 months increased by 37% in those with RDW levels of 15.1%–29.9% (tertile 3) in the multivariable-adjusted model. *P*-values for the trend tests were all less than 0.05, indicating that our findings were robust.

**Table 3:** Association of red cell distribution width with the risk of readmission within 3 months.

RDW, %	No. of events	The risk of 3-month readmission, HR (95% CI), *P*-value		
		Non-adjusted model	Minimally adjusted model	Multivariable adjusted model
Per 1% increment	495(25.0%)	1.07 (1.02, 1.11) 0.0015	1.06 (1.02, 1.11) 0.0025	1.09 (1.03, 1.15) 0.0039
Tertile				
Tertile 1 (11.8–13.8)	132(20.8%)	Ref	Ref	Ref
Tertile 2 (13.9–15.0)	175(26.2%)	1.29 (1.03, 1.62) 0.0276	1.27 (1.01, 1.59) 0.0392	1.19 (0.92, 1.54) 0.1952
Tertile 3 (15.1–29.9)	188(27.9%)	1.38 (1.10, 1.72) 0.0049	1.35 (1.08, 1.68) 0.0090	1.37 (1.03, 1.83) 0.0307
P for trend		0.006	0.010	0.035

HR, hazard ratio; CI, confidence interval; RDW, red cell width distribution; Ref., reference; BMI, body mass index; CCI, charlson comorbidity index; NYHA, new york heart association; BNP, brain natriuretic peptide; RBC, red blood cell; HB, hemoglobin; ALB, albumin; ALT, alanine aminotransferase; LDH, lactate dehydrogenase; eGFR, estimated glomerular filtration rate.

Non-adjusted model: no adjustment for covariates.

Minimally adjusted model: adjusted for age and, sex.

Multivariable adjusted model: adjusted for age, sex, BMI, CCI score, NYHA cardiac function classification, type of heart failure, BNP, RBC, HB, total protein, ALB, ALT, LDH, and eGFR.

### 3.4. Dose-response relationship between RDW and the risk of readmission within 3 months

**Figure 1:**
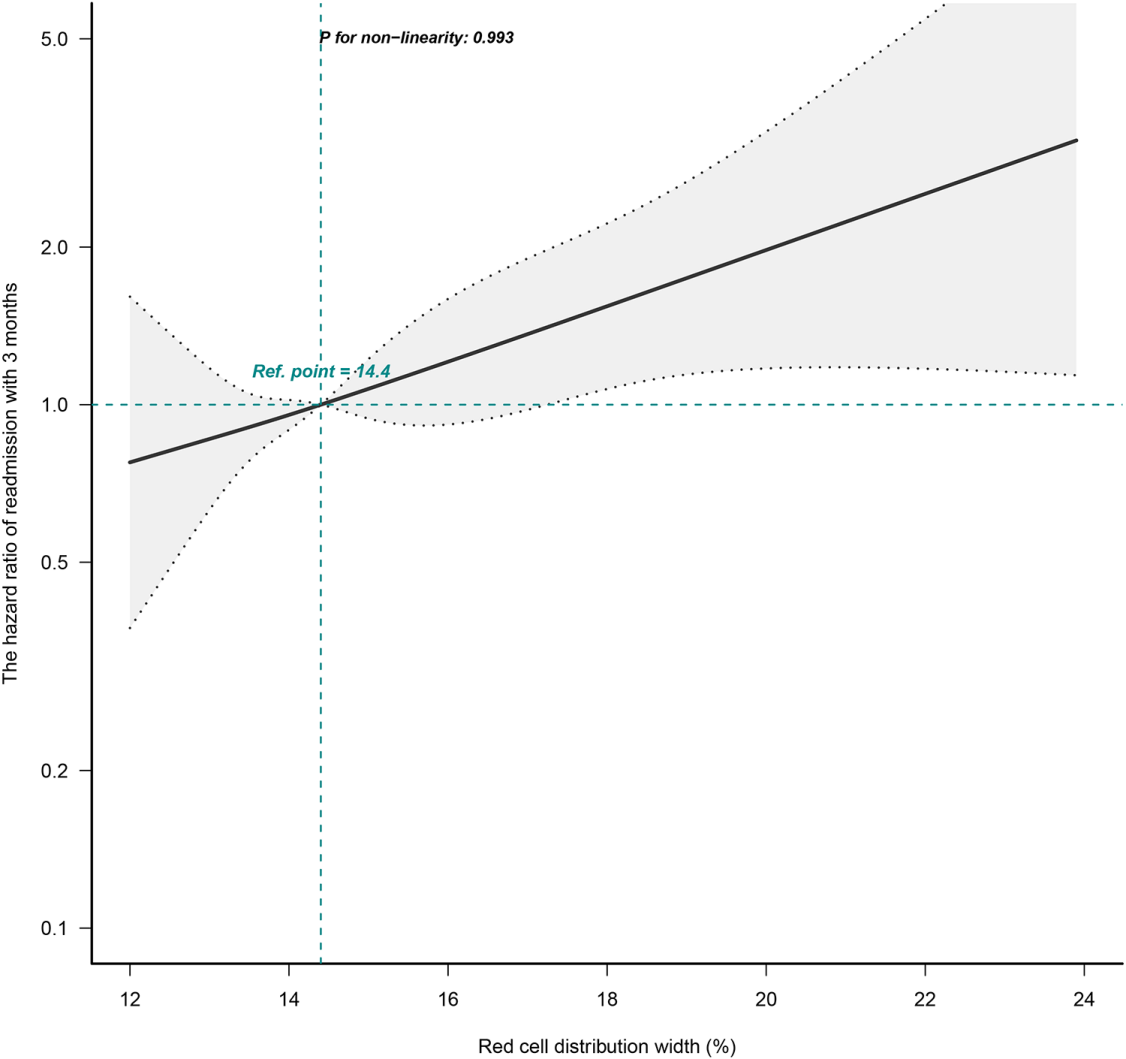
Dose-response relationships between RDW and the hazard ratio of readmission within 3 months. The black solid line represents the estimated risk of readmission within 3 months, with dashed lines showing 95% confidence intervals. The blue histogram at the bottom represents the distribution of RDW. Analyses were adjusted for age, sex, BMI, CCI score, NYHA cardiac function classification, type of heart failure, BNP, RBC, HB, total protein, ALB, ALT, LDH, and eGFR. RDW, red cell distribution width; BMI, body mass index; CCI, charlson comorbidity index; NYHA, new york heart association; BNP, brain natriuretic peptide; RBC, red blood cell, HB, hemoglobin; ALB, albumin; ALT, alanine aminotransferase; LDH, lactate dehydrogenase; eGFR, estimated glomerular filtration rate.

The correlation between RDW and the risk of readmission within 3 months was evaluated on a continuous scale using smoothed curve fitting (restricted cubic spline method) based on the Cox proportional hazards models. The fully adjusted smoothed curve fitting showed a linear association between RDW and the risk of readmission within 3 months, with a *P*-value for non-linearity of 0.993 (Figure 1). When the RDW was greater than 14.4%, the HR of readmission within 3 months for patients with HF was greater than 1.

### 3.5. Subgroup analysis

**Figure 2:**
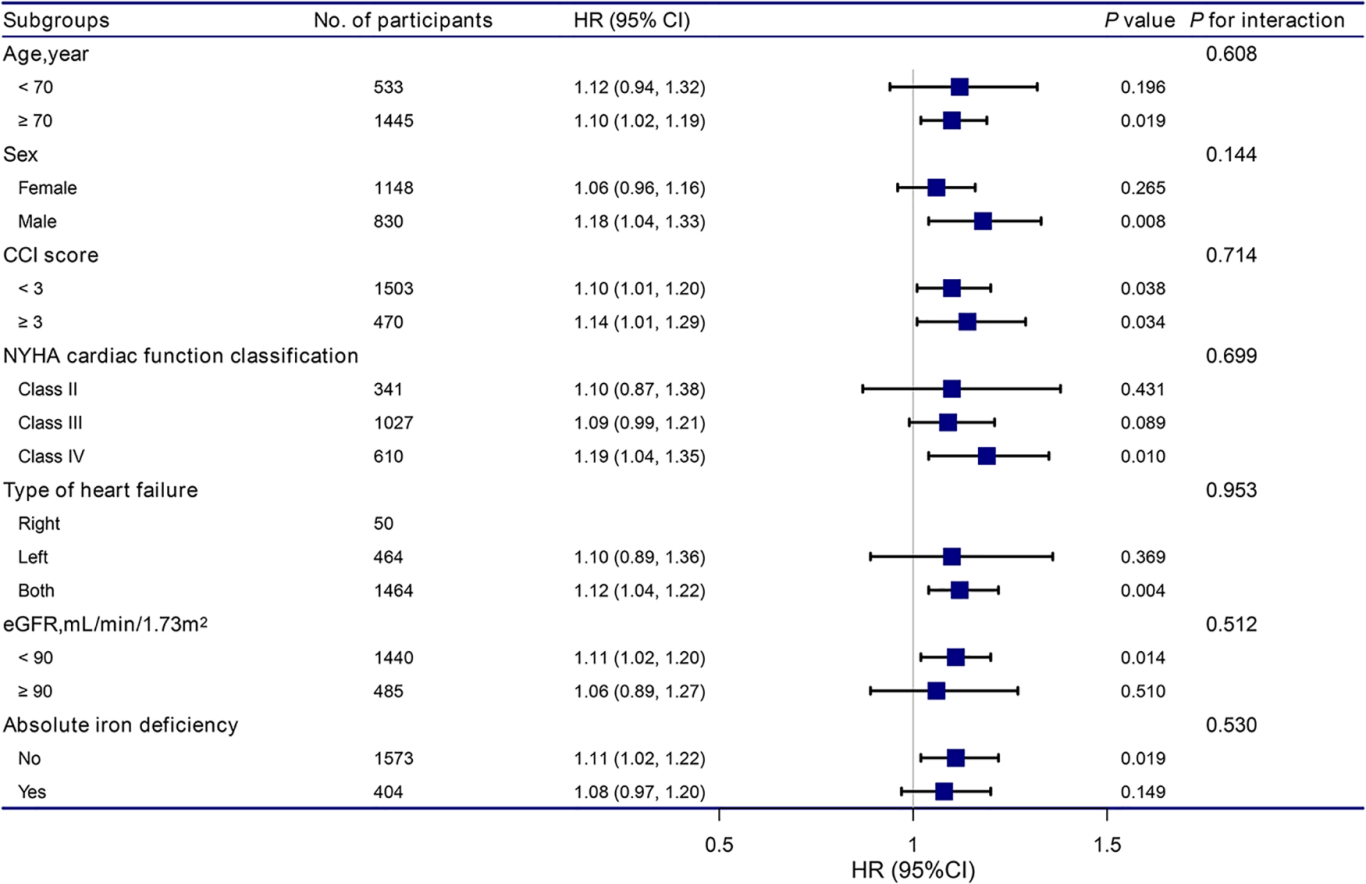
Association between RDW and the hazard ratio of readmission within 3 months according to subgroup. Analyses were adjusted for age, sex, BMI, CCI score, NYHA cardiac function classification, type of heart failure, BNP, RBC, HB, total protein, ALB, ALT, LDH, eGFR, and absolute ID, except for the stratification variable. RDW, red cell distribution width; BMI, body mass index; CCI, charlson comorbidity index; NYHA, new york heart association; BNP, brain natriuretic peptide; RBC, red blood cell, HB, hemoglobin; ALB, albumin; ALT, alanine aminotransferase; LDH, lactate dehydrogenase; eGFR, estimated glomerular filtration rate.

The stratiﬁcation and interaction analyses of the correlation between RDW and the risk of readmission within 3 months for patients with HF are shown in Figure 2. The results of the subgroup analysis were highly consistent with those of the multivariate Cox regression analysis. Interaction analysis results showed no interactive roles in the subgroup.

### 3.6. Kaplan–Meier survival curve

**Figure 3:**
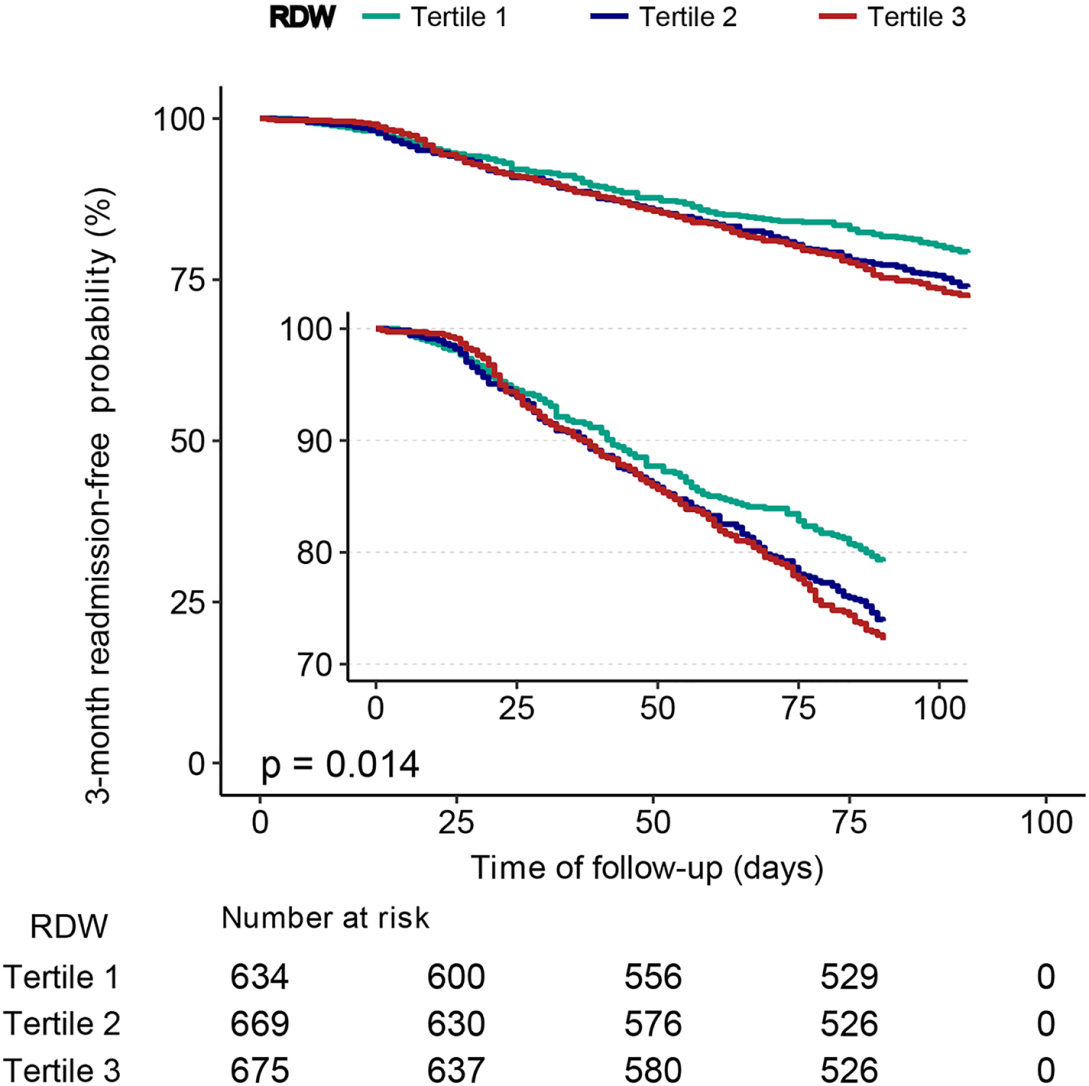
Kaplan–Meier curves for 3-month readmission-free probability in the patients with heart failure for categories of RDW tertiles. RDW, red cell distribution width.

Patients with a lower RDW (tertile 1) had a significantly higher 3-month readmission-free probability than those with a higher RDW (tertiles 2 and, 3) (*P* = 0.014), as shown in Figure 3.

## 4. Discussion

This retrospective cohort study focused on determining the association between RDW and the risk of readmission within 3 months in patients with HF. It suggested that RDW was linearly and positively associated with the risk of readmission within 3 months in middle-aged and elderly patients with HF in China after adjusting for several major confounding factors. In the multivariable-adjusted model, we found that every 1% increase in RDW was correlated with a 9% increased risk of readmission within 3 months (HR = 1.09, 95% CI: 1.03–1.15, *P* < 0.005) and the highest RDW group (tertile3) had a higher risk of 3-month readmission compared with the lowest group (tertile1). These results were consistent for each subgroup.

RDW is strongly associated with the prognosis of patients with HF. Consistent with our results, previous studies have shown that the RDW at admission is associated with a multifactorial adjusted risk of rehospitalization. Remo et al. showed that patients with high RDW had a risk ratio of 1.55 (95% CI: 1.08–2.22) for death or reoccurrence of HF from any cause compared with patients with a low RDW (20). Kyoung et al. found that readmission rates for older patients were higher in the highest group of the RDW category (7.4% in the lowest group and 15.8% in the highest group, *P*-trend < 0.001) (21). However, some studies have reported inconsistent results. Tan et al. included patients with HF in China by using multivariate logistic regression models to predict the risk of 3-month readmission and concluded that simple observation of RDW from clinical blood test reports could not be used to visually determine the risk of readmission (22); the area under the red cell volume distribution width curve also confirmed its weaker discriminatory ability (C statistic = 0.61); however, their study included was only 350 patients, and the study population was limited to the HF population in central China. Our study had a larger sample size, and the patients were not limited to central China to further investigate the relationship between RDW and the risk of readmission within 3 months.

RDW is a common clinical measure of heterogeneity in RBC size (23). A higher RDW indicates a defect in the maturation or degradation of blood cells (24). An elevated RDW has been shown to be associated with anemia, inflammatory diseases (e.g., infections and tumors), chronic kidney disease, diabetes mellitus, and cardiovascular diseases (e.g., HF, hypertension, stroke, and atrial fibrillation) (25–30). However, the specific mechanisms underlying the association between RDW and poor prognosis in chronic diseases, including HF, are not fully understood. Multiple interrelated pathological mechanisms, including nutritional deficiency, ineffective erythropoiesis, reduced iron mobilization, oxidative stress, chronic inflammation, adrenergic stimulation, and enhanced immune system activation, are thought to be associated with an increase in RDW and poor prognosis in HF patients (31, 32). The main role of erythrocytes is to transport oxygen and carbon dioxide from the lungs to the tissues and to maintain the acid-base balance of the body (33). Recent studies suggest that erythrocytes are involved in nitric oxide metabolism and have secretory functions (release of nitric oxide, nitric oxide metabolites, and adenosine triphosphate) (33). The mediators released by erythrocytes play a key role in cardiovascular regulation and have a direct impact on the heart. Nutritional deficiencies lead to inadequate erythropoiesis and increased RDW (34). Nutritional factors are critical for improving the medium-to-long-term prognosis of patients with HF. Chronic inflammation and oxidative stress can lead to increased RDW by suppressing effective bone marrow erythropoiesis and increasing RBC variability (35). On the one hand, inflammatory markers (interleukin-6 and tumor necrosis factor-α) disrupt erythropoiesis by directly inhibiting erythroid precursors, affect iron metabolism, and reduce erythropoiesis; it also decreases erythropoietin secretion from the kidney (36, 37). On the other hand, oxidative stress decreases erythrocyte survival and leads to increased circulating premature erythrocytes, resulting in anisocytosis and increased erythrocyte lysis, which leads to increased free radicals and adverse effects on the heart (38, 39). Thus, higher oxidative stress is another potential mechanism linking RDW to a poor prognosis in patients with HF.

Based on the subgroup analysis, we found that the association between RDW and the risk of readmission within 3 months was stronger in men than in women (HR: 1.18 vs. 1.06). Sex differences exist in almost all aspects of HF. First, there are differences in cardiac anatomy between men and women, with women without heart disease having a higher left ventricular ejection fraction than men (40). Women with HF with preserved ejection fraction have smaller, stiffer hearts and more frequent concentric remodeling than men with HF with preserved ejection fraction (41). Second, from a pathophysiological point of view, women with HF with reduced left ventricular ejection fraction have better adaptation of the myocardium to stress and a lower risk of ventricular tachycardia, atrial fibrillation, and sudden cardiac death than men (42, 43). Third, several neurohormonal modulators have been shown to have better therapeutic effects in women with HF, but even before the recent introduction of neurohormonal modulators into HF treatment, clinical outcomes for women with HF with preserved or reduced ejection fraction were consistently better than for men (44). Mechanistic studies to explain these sex-related differences in HF treatment are lacking, and further research is required to verify these phenomena. An analysis of PARADIGM-HF suggested that mortality and hospitalization rates are lower in women for reasons that are not yet clear (45). Moreover, future consideration of gender-based differences in treatment is needed to reduce the risk of readmission.

In the subgroup analysis, we found that the association between RDW and the risk of readmission within 3 months was stronger among patients with more co-morbidities (CCI score ≥3) and poor cardiac function (NYHA cardiac function class IV). Previous studies have demonstrated that co-morbidities are common in hospitalized elderly patients and are associated with long-term prognosis (46). Moreover, other studies have documented co-morbidities and cardiac function class as independent adverse prognostic factors in elderly patients with acute HF (47), consistent with our results.

Our study has the following strengths. First, this study had a larger sample size than previous studies. Second, the effect modification factor analysis allowed better use of the data to draw stable conclusions across different subgroups. However, this study has some limitations. First, because this was an observational study, we could not confirm a causal relationship between RDW and the risk of readmission within 3 months. Second, this study only assessed RDW values at the first admission. Therefore, it is impossible to evaluate the influence of dynamic changes in RDW on the risk of readmission within 3 months. Third, the subjects of this study were all patients with HF in China; therefore, there are some deficiencies in the extrapolation and generalizability of the study.

In conclusion, we found that RDW was positively and linearly related to the risk of readmission within 3 months in patients with HF. Patients with HF with higher RDW values also had a higher risk of readmission within 3 months. Further studies on the ability of RDW to predict the risk of readmission are needed.

## Data Availability

The datasets presented in this study can be found in online repositories. The names of the repository/repositories and accession number(s) can be found below: https://doi.org/10.13026/8a9e-w734.
